# Clinical application of 3D printing technology to the surgical treatment of atlantoaxial subluxation in small breed dogs

**DOI:** 10.1371/journal.pone.0216445

**Published:** 2019-05-03

**Authors:** Hiroaki Kamishina, Taku Sugawara, Kohei Nakata, Hidetaka Nishida, Naoko Yada, Toru Fujioka, Yoshihiko Nagata, Akio Doi, Naoyuki Konno, Fujio Uchida, Sadatoshi Maeda

**Affiliations:** 1 Joint Department of Veterinary Medicine, Faculty of Applied Biological Sciences, Gifu University, Gifu, Japan; 2 The United Graduate School of Veterinary Sciences, Gifu University, Gifu, Japan; 3 Center for Highly Advanced Integration of Nano and Life Sciences, Gifu University, Gifu, Japan; 4 Department of Spinal Surgery, Research Institute for Brain and Blood Vessels Akita, Akita, Japan; 5 KOIWAI Co., Ltd., Odawara, Japan; 6 Iwate Prefectural University, Takizawa, Japan; 7 Akita Precisions Industry Co., Ltd., Daisen-shi, Akita, Japan; 8 Akita Industrial Technology Center, Akita, Japan; University of Bologna, ITALY

## Abstract

Atlantoaxial instability (AAI)/subluxation commonly occurs in small breed dogs. Ventral stabilization techniques using screws and/or pins and a plate or, more commonly, polymethylmethacrylate are considered to provide the most favorable outcome. However, the implantation of screws of sufficient sizes for long-term stability becomes challenging in toy breed dogs (e.g. <2 kg). We herein report the application of 3D printing technology to implant trajectory planning and implant designing for the surgical management of AAI in 18 dogs. The use of our patient-specific drill guide templates resulted in overall mean screw corridor deviations of less than 1 mm in the atlas and axis, which contributed to avoiding iatrogenic injury to the surrounding structures. The patient-specific titanium plate was effective for stabilizing the AA joint and provided clinical benefits to 83.3% of cases (15/18). Implant failure requiring revision surgery occurred in only one case, and the cause appeared to be related to the suboptimal screw-plate interface. Although further modifications are needed, our study demonstrated the potential of 3D printing technology to be effectively applied to spinal stabilization surgeries for small breed dogs, allowing for the accurate placement of screws and minimizing peri- and postoperative complications, particularly at anatomical locations at which screw corridors are narrow and technically demanding.

## Introduction

Atlantoaxial instability (AAI) has been widely described in the veterinary literature. Although a few studies have been conducted on medium to large breed dogs, AAI predominantly occurs in small breed dogs (i.e. less than 5 kg) [1]. Most predisposing factors in small breed dogs are congenital or developmental in nature including abnormalities of the atlas, axis, or ligaments attaching these two vertebrae or those attaching the dens and occipital bone [2–4]. Aplasia or hypoplasia of the dens or non-union of the dens with the vertebral body of the axis is frequently reported in symptomatic cases. Dogs are typically younger than one year of age when the first clinical signs develop if the underlying etiology is congenital or developmental. Intermittent stiffness or screaming suggestive of varying severities of neck pain or discomfort, ambulatory or non-ambulatory tetraparesis, and other neurological deficits are typically observed in affected dogs [1, 5]. Sudden death from respiratory arrest may also occur if further mechanical forces cause the unstable atlantoaxial (AA) joint to dislocate significantly.

Although non-surgical, conservative management is selected for immature and/or high-risk patients [6], surgical treatment is indicated in general to stabilize AAI [7]. Several surgical techniques for AAI have been described in the veterinary literature and may be grouped into ventral or dorsal fixation. Ventral fixation is generally considered to be superior to dorsal fixation due to the better visualization of the AA junction, thereby allowing the inspection of the dens, the removal of a malformed dens, and better reductions in the AA joint. Ventral fixation is also preferred because bone fusion (ankylosis) may be achieved by cancellous bone graft placement in the AA joint space after the removal of articular cartilage. In most cases, the atlas and axis are anchored by screws, threaded pins, or Kirschner wires and supported by polymethylmethacrylate (PMMA) or plating [4, 5, 8, 9].

Although current surgical techniques provide a modest success rate, surgical complications, if they occur, are associated with a high mortality [4, 10]. Most complications are associated with the misplacement of implants, resulting in iatrogenic injury to the arteries, venous sinus, nerve roots, and dural sac, or implant failure due to the suboptimal location or undersizing in implants due to small safe implant corridor and consecutive implant failure. Even though the detailed incidence of complications has not been published, the overall success rate of ventral fixation techniques markedly varied between 44 and 90% [1, 3, 4, 7, 10], and the reported mortality rate was between 10 and 39% [1, 3, 4]. Therefore, the accurate placement of implants is imperative for avoiding perioperative complications and achieving long-term success in the treatment of AAI. These prerequisites become especially challenging for toy breed dogs such as those less than 2 kg.

In order to establish a safer surgical technique for AAI in small breed dogs, we utilized 3D printing technology. We herein describe our custom-made drill guide template system, which allows for the accurate placement of screws and a custom-made titanium plate for AA fixation.

## Materials and methods

### Ethics statement

This research was conducted as a prospective multi-institutional clinical study performed on client-owned small breed dogs with AAI. Surgical procedures were performed at either private hospitals with informed consent from the owners or a university teaching hospital with informed consent from the owners and under the approval of the Institutional Animal Care and Use Committee of Gifu University (Approval number: 15082). All procedures were performed in accordance with the guidelines regulating animal use and ethics at Gifu University.

### Cases

Dogs diagnosed as AAI by means of CT and MRI between January 2014 and June 2016 and underwent atlantoaxial fixation surgery were included in this study. Dogs that had lost to follow-up by 1 year post-surgery were excluded. All cases were diagnosed with AAI based on radiography followed by computed tomography (CT) (Alexion Advance, Toshiba or ECLOS-8S, Hitachi Medical systems) (120kV, 100mA, eff.mAs80, FOV120.0) and magnetic resonance imaging (MRI) (0.4-Tesla APERTO Eterna, Hitachi, or AIRIS Vento LT, Hitachi Medical systems). Age and body weight were recorded at each visit. Neurological statuses were graded as previously reported [10]. A neck brace was applied to all cases until surgery. A low dose of prednisolone (0.5 mg/kg/24 h) was administered as needed to cases exhibiting severe neurological deficits such as non-ambulatory tetraparesis or tetraplegia.

### Imaging and drill guide template production

**Fig 1:**
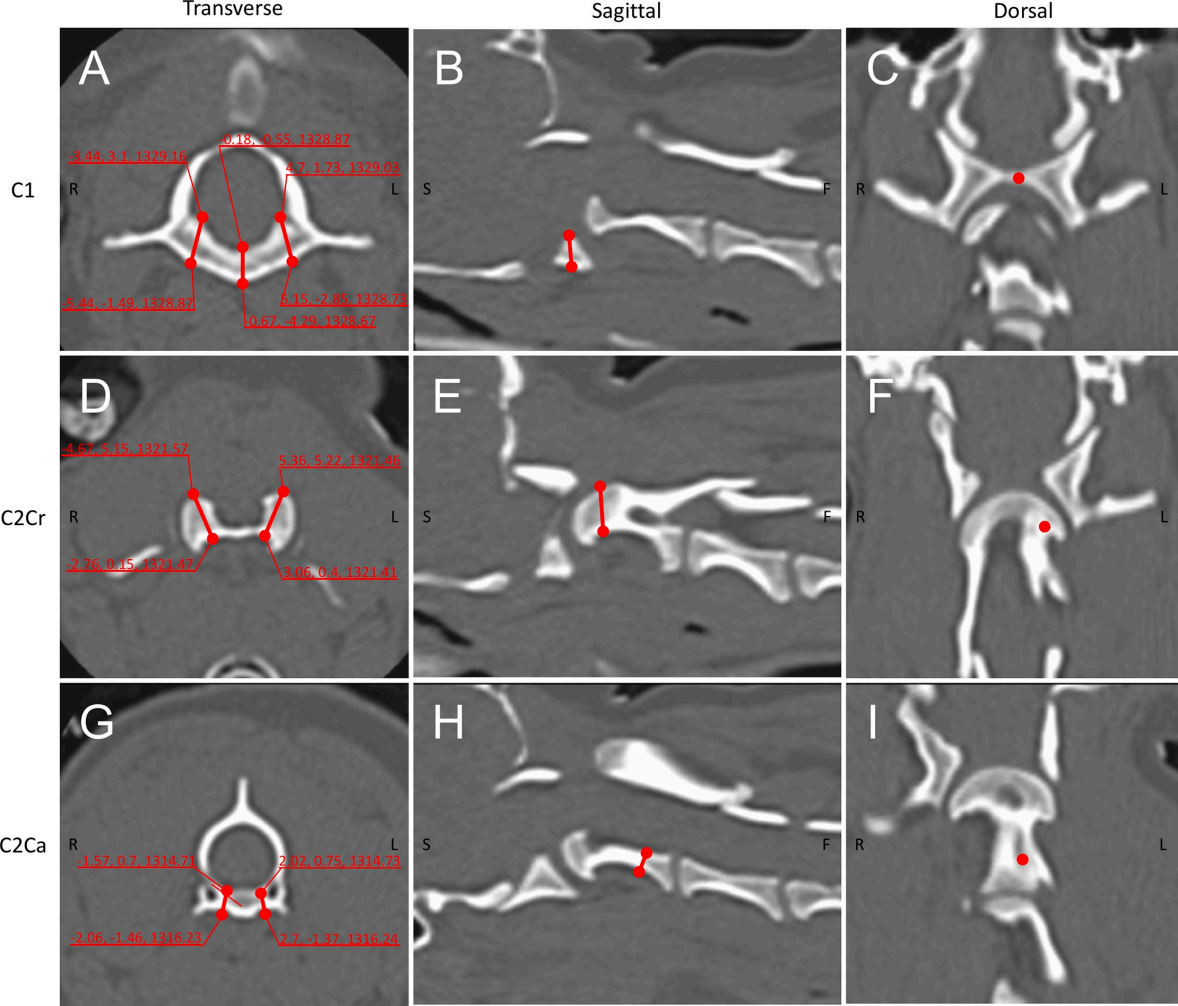
Preoperative planning of screw trajectories. The trajectories and the coordinates for bone entry points and the tips of screws were obtained for the atlas (A, B, C), cranial axis (D, E, F), and caudal axis (G, H, I). The trajectories of each screw were assessed in a 3D manner using multiplanar reconstructed images.

The high-accuracy 3D CT scans described above with a slice thickness of 0.5–1.0 mm were used to obtain preoperative images of the upper cervical spine. In cases under development (defined as those < 8 months old), CT scanning was delayed until they reached at least 8 months old. CT data of other cases were obtained on the day of the first visit. Images were exported in the DICOM format to 3D/multiplanar imaging software (Ziostation, Ziosoft). The trajectories of the screws in the atlas and axis were decided on reconstructed bone images viewed on several optional planes (Fig 1). In the atlas, trajectories were planned as previously described with some modifications [10]. The first screw was set to penetrate the midline of the ventral arch, at the midpoint of the rostro-caudal direction. The planned surgical fixation included two additional lateral screws into the ventral arch with a slight medial angle. Three screws inserted to C1 were placed bicortically. In the axis, 4 screws were planned for insertion as previously described with some modifications [7, 11]. The location of cranial screws was set in the thickest part of the vertebral body. The locations of the tips of these cranial screws were set to penetrate the transcortex of the vertebral body in order to achieve a bicortical purchase. The caudal screws were set to be inserted into the thick part of the caudal vertebral body. The locations of the screw tips were planned to be close to the transcortex of the vertebral body, but not to perforate the cortex. The coordinates of the bone entry points and tips of the screws were recorded (Fig 1). The diameter and length of the screws were selected based on a computer simulation adjusting the width of the ventral arch of C1 or vertebral body of C2.

**Fig 2:**
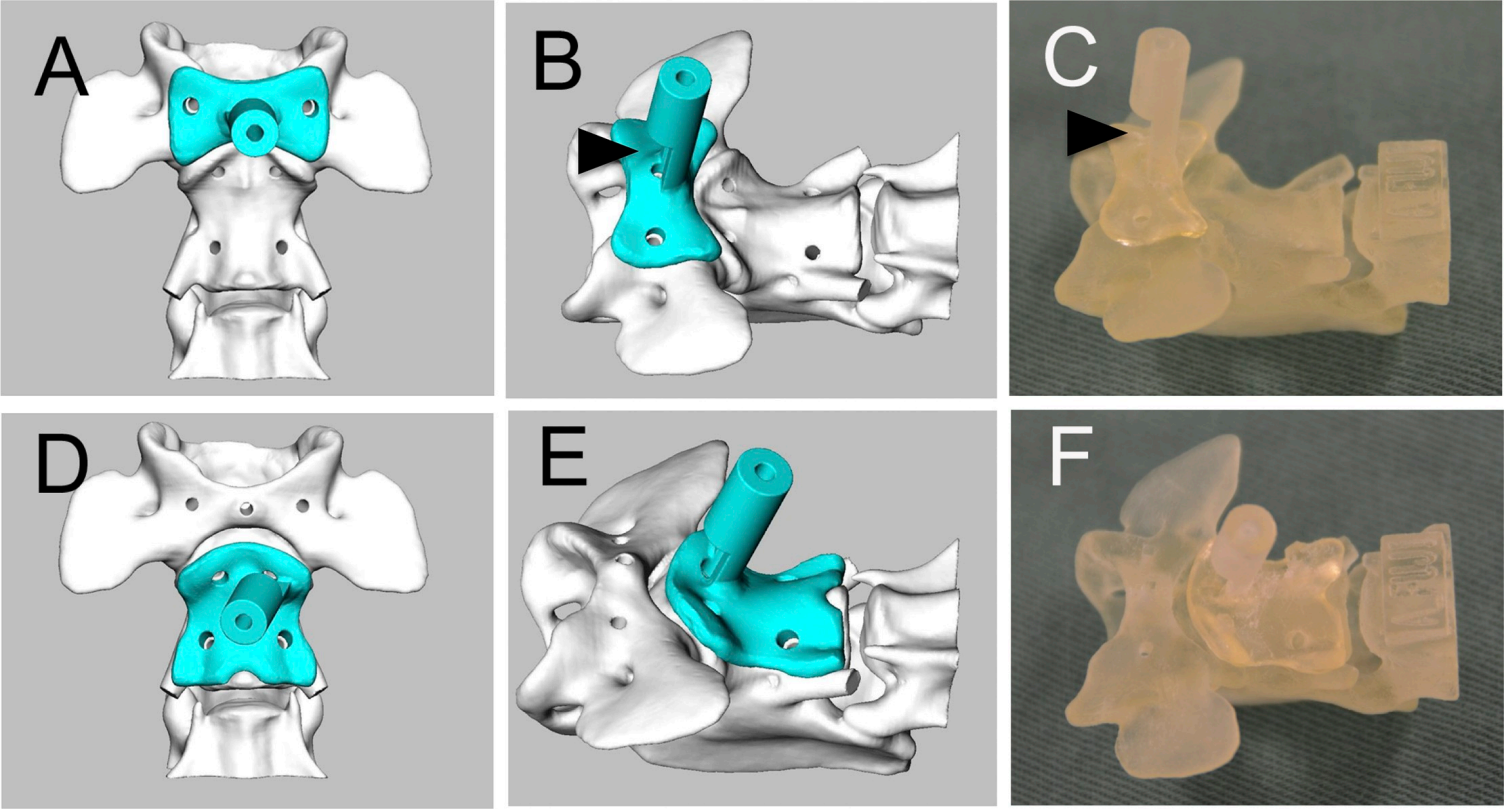
Design and fabrication of patient-specific drill guide templates. Drill guide templates for the atlas (A and B) and axis (D and E) were designed using 3D modeling software. Contact between the fabricated drill guide templates and bone models was assessed preoperatively (C and F). An arrowhead in B and C shows the location of an opening at the base of the cylindrical sleeve.

Drill guide templates were designed for each case in order to achieve the accurate guidance of the screws. The design of drill guide templates involved two steps. Bone data were extracted from CT data in the DICOM format using image processing software (VG Studio Max; Volume Graphics GmbH or Mimics; Materialize). The range of Hounsfield units (HU) to be extracted was set to only extract bone data and this was further adjusted through visual inspections of transverse images of the atlas and axis. Bone data were then transferred to 3D modeling software (Freeform; 3D Systems, Inc.), the atlas and axis were separated, and the platforms of the drill guide templates that fit the surfaces of each bone were designed (Fig 2A, 2B, 2 and 2E). Cylindrical sleeves (internal diameter of 1.2 mm), the angles of which were adjusted to the predetermined coordinates, were attached to the platform that fit and locked the patient-specific 3D shape of the ventral surfaces of C1 and C2. Templates were fabricated from non-soluble acryl using a 3D printing system (Connex500, Stratasys Ltd.) (Fig 2C and 2F). Layer thickness was set at 150 μm. Small windows were made in the cylindrical structures of the drill guide templates in order to confirm that drilling was performed exactly through the entry points (Fig2 B, C). Patient-specific 3D bone models of the upper cervical spine were also made with the same 3D printing system in order to confirm the fit of the templates to the ventral surfaces of C1 and C2 (Fig 2C and 2F). In each case, a simulation of screw placement was performed before surgery using drill guide templates and the corresponding bone models.

### Design and fabrication of custom-made titanium plates

**Fig 3:**
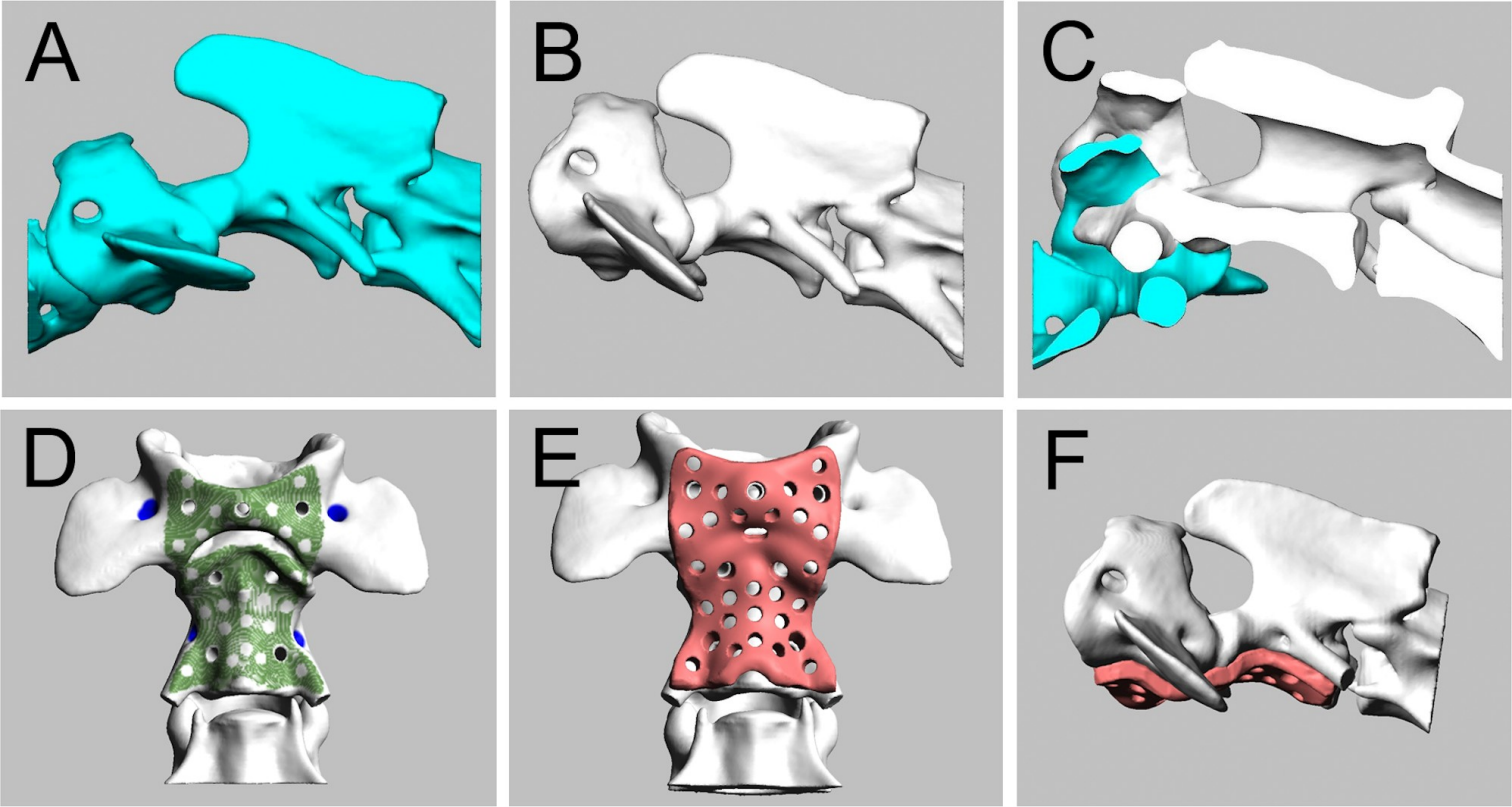
Simulation of AA joint realignment and titanium plate designing. Using 3D modeling software, the luxated AA joint (A) was realigned to a normal anatomical relationship (B). This was typically achieved by moving the atlas dorsally in order for the ventral arch to make contact with the dens (C). On the realigned AA joint, the surface area of the ventral arch and axial vertebral body that makes contact with the plate was examined (D) and a 1.5-mm-thick plate was designed (E and F).

Using reconstructed 3D images on modeling software (Freeform; 3D Systems, Inc.), a luxated AA junction was reduced to the normal position (Fig 3A and 3B). In cases with an intact or nearly intact dens, the relationship between the ventral arch of the atlas and the dens of the axis was restored, referring to the mid cross-sectional image of the sagittal plane (Fig 3C). In cases with an aplastic or severely hypoplastic dens, the ventral surfaces of the atlas and axis were aligned in order to maintain the vertebral canal. We also ensured that the positions of the atlas and axis on the dorsal plane were aligned. Once the realigned position was set, the surface area of the atlas and axis which is covered by the titanium plate was determined (Fig 3D). AA plates with a thickness of 1.5 mm were designed to have screw holes that fit the predetermined locations and angles (Fig 3E and 3F). The AA plates also had 1-mm pores for bone invasion, which was selected based on our previous study (unpublished data). Titanium plate printing was performed on a 3D printer (Arcam A2x; Arcam AB) using Grade23 titanium Ti6Al4V powder (Arcam AB), which contains O_2_ <0.13% as the printing material. Electron beam melting was operated at an ambient temperature of 730°C under a vacuum of less than 2×10^−2^ mbar and an acceleration voltage of 60 kV. The beam diameter was approximately 300 μm and the layer thickness was 50 μm, resulting in a maximum printing accuracy of approximately ±200 μm.

### Surgery

All surgical rehearsals and actual procedures were performed by the same individual (HK). All dogs were anesthetized using a standard protocol and maintained with isoflurane in oxygen. Dogs were positioned in a dorsal recumbency on a vacuum beanbag with the neck slightly elevated and extended. The surgical area was prepared and draped, including the caudal mandible and right proximal humerus. In most cases, autologous cancellous bone was harvested from the humerus for bone grafting; however, femurs were also used in some dogs. The approach to the AA junction was performed as described previously [9]. The longus colli muscles were elevated and the AA junction was exposed after capsulotomy. Articular cartilage was removed with a round burr and bone curette.

Drill guide templates were sterilized with an ethylene oxide gas sterilizer and the titanium plates were autoclaved. Specifically, the drill guide template was firmly attached to the ventral surface of C1 or C2 by manually pressing it to the bone using a mosquito forceps, and drill holes were made using Kirschner wires (0.9 mm for 1.2-mm cortical screws or 1.2 mm for 1.7-mm cortical screws). We determined the appropriate depth of the drill hole based on a preoperative CT image. The Kirschner wire was held with a wire collet at a predetermined position so that the wire only passes through the cylindrical drill sleeve and penetrates the desired depth of the bone. After all screw holes had been drilled, the autologous bone graft was placed in the AA joint space and a titanium plate was fixed with the predetermined diameters of titanium cortical screws (U-CFM screws, Stryker, MI, USA). After our first few cases, we noted that using the drill guide templates for the center screw hole of C1 and cranial two screw holes of C2 only made the remaining procedure easier. We made the above decision for the following reasons: 1) even though the drill guide templates were custom-made and, in theory, perfectly fit the vertebral surface, there was potentially a slight deviation between the guide and bone, particularly if the target bony surface lacked asperity, which normally helps engagement of the guide to the bone, 2) slight deviations in multiple guide-bone interfaces may potentially result in marked misalignment and preclude the insertion of some screws, and 3) once the plate was secured by the first screw, the remaining screw holes may be drilled through the screw holes of the plate with high precision. The longus colli muscles were apposed over the implants, and the remainder of the wound was closed in a routine manner.

The locations of the screws were examined on postoperative CT scans. Deviations in screw trajectories were assessed using image analysis software (Virtual Place; Aze Ltd.). The DICOM data of preoperative and postoperative CT were imported into the software, the atlas and axis were visually superimposed individually, and the 3D coordinates of the implanted screw locations on preoperative CT images were identified. Deviations in the coordinates of the entry points and exit points between pre- and postoperative images on the transverse, dorsal, and sagittal planes were assessed. In addition, complications related to screw insertion were recorded intraoperatively and by assessing postoperative radiographs and CT images. Specifically, major intraoperative complications were defined as vertebral fracture, the violation of vital structures (the vertebral canal, spinal cord, spinal nerves, internal vertebral venous plexus, and vertebral artery), and screw/plate dislocation were recorded. We designed screw coordinates for the atlas as bicortical engagement. Therefore, the violation of the spinal canal of the atlas by the inserted screws was not considered to be a complication unless the screws impinged on the cord. We also evaluated the bone mineral density (BMD) [12] and cortical thickness [13] of C1 according to previously reported methods with some modifications. Specifically, CT images were viewed on medical image archiving and communication platform software (Osirix Foundation, V4.1.2 Geneva, Switzerland) and HU calculations were performed. Elliptical regions of interest (ROI) for HU value measurements were drawn on transverse images of preoperative CT scans at three locations for C1 and four locations for C2, which were adjusted to include the actual corridors of screw insertion for each dog, using postoperative CT images as a reference. Similarly, cortical thickness was measured from the outer surface of the cortex to the inner surface of the cortex of C1 at the postoperative screw corridors. In order to avoid measurement bias, all measurements were performed by a single observer (KN) unaware of the status of the operated dogs. The HU calculations, as a representation of BMD, and cortical thickness measurements were performed postoperatively.

### Follow-up evaluation

Perioperative (2 weeks), short-term (3 months) postoperative, and mid-term (12 months) postoperative follow-up evaluations consisted of physical and neurological examinations. At each visit, lateral and ventrodorsal views of plain radiographs were taken to evaluate if any implant failure was present (i.e. screw loosening, plate dislocation, and screw or plate breakage). In 4 cases (case No.4, 6, 14, 18), a telephone interview was performed in order to assess short-term and/or long-term outcomes. Neurological grading was only performed when dogs were assessed directly by veterinarians. If postoperative complications were noted, potential predisposing factors such as age, body weight, neurological grades on the day of surgery, the degree of deviations in the inserted screws, vertebral HU, and cortical thickness were statistically assessed between the complication and non-complication groups.

### Statistical analyses

All statistical analyses were performed with the Wilcoxon rank-sum test by JMP®10 (SAS Institute Inc., U. S. A). The level of significance was set at p < 0.05.

## Results

### Case population

We performed AA fixation on 18 dogs. None of the dogs were excluded from this study. Seven dogs were male, 2 of which were castrated, and 11 dogs were female, 4 of which were spayed. Median age at onset was 28.5 months (range, 2–93 months), while that at surgery was 35 months (range, 8–101 months). Median body weight at surgery was 2.22 kg (range, 1.3–5.58 kg). AA anomalies were identified on CT images in 13 dogs; a hypoplastic dens was present in 8 dogs and non-union dens in 4 dogs, and 1 dog had a hypoplastic dens that was non-union. In 4 dogs, an obvious history of trauma was present that appeared to trigger the clinical onset. Three of these dogs had a hypoplastic dens. Clinical information and the imaging findings of all cases were summarized in Table 1.

**Table 1:** Clinical information and findings of axial.

							Neurological grade							
Case No.	breed	body weight (kg) at surgery	sex	age at onset (month)	age at surgery (month)	potential predisposing factor(s)	1st visit	pre-surgery	perioperative period	short follow-up period	mid follow-up period	Last follow-up period (month)	complications	surgery time (min)
1	Toy poodle	2.65	SF	93	101	trauma (hypoplastic dens)	3	3	4	4	4	22	screw loosening of C1	93
2	Maltese	2.22	F	45	49	trauma	2	4	4	4	4	22	screw loosening of C1	111
3	Pomeranian	1.48	F	5	8	hypoplastic dens	4	5	5	5	5	17		121
4	Chihuahua	1.78	F	66	69	hypoplastic dens	3	4	4	5	5	47	screw loosening of C1	235
5	Miniture Dachshund	3.12	SF	4	17	non-union dens	3	4	4	5	5	31		159
6	Shih Tzu	5.58	M	10	18	non-union dens	1	3	4	5	5	27		228
7	Chihuahua	3.45	M	82	93	ND	2	4	4	5	-	died 11mon post-surgery	screw loosening of C1	290
8	Pomeranian	2.85	M	5	8	trauma (hypoplastic dens) non-union	4	4	5	5	5	41	screw loosening of C1	245
9	Toy poodle	1.3	F	9	15	trauma (hypoplastic dens)	2	5	5	5	5	23		188
10	Chihuahua	1.8	M	12	17	ND	4	5	5	5	5	25		121
11	Toy poodle	4.66	CM	64	69	non-union dens	2	4	5	5	5	25		242
12	Toy poodle	2.22	F	2	12	hypoplastic dens	4	4	5	5	5	22		142
13	Toy poodle	2.1	SF	55	57	hypoplastic dens	4	5	5	5	5	45		287
14	Chihuahua	2.2	F	6	21	ND	2	4	4	5	5	24		130
15	Toy poodle	2.24	SF	50	54	hypoplastic dens	5	5	5	5	5	23		101
16	Toy poodle	1.82	F	7	15	hypoplastic dens	4	4	5	5	5	17		103
17	Pomeranian	2.08	M	50	61	C1-2 overlapping	2	4	3	-	-	9		156
18	Mix	4.15	CM	69	73	non-union dens	2	4	4	4	4	17	screw loosening of C2 Plate disloation	139

Neurological grades were as follows: 5, normal gait with or without neck pain only; 4, ataxia; 3, ambulatory tetraparesis; 2, non-ambulatory tetraparesis; 1, tetraplegia.

SF, spayed female; F, intact female; CM, castrated male; M, intact male. ND, not determined

### Surgery

**Fig 4:**
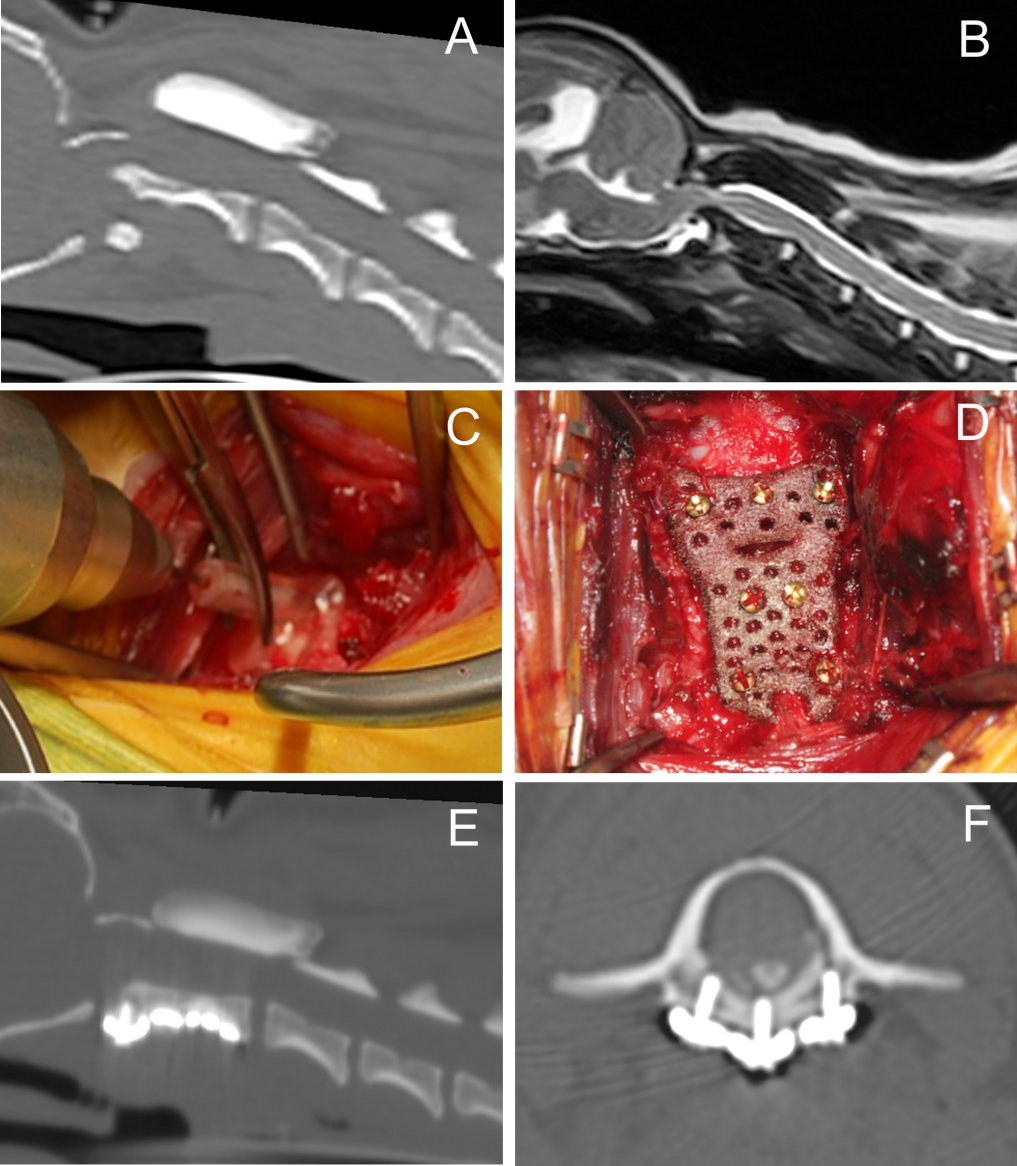
Illustration of a representative case (Case No.2). Preoperative CT (A) and MR (B) images demonstrating AA subluxation and spinal cord compression by the dorsally deviated dens. The dens appeared to be slightly hypoplastic, and, thus, may have been predisposed to AAI. Spinal fracture was not detected on CT images. During surgery, the drill guide template was firmly attached to the ventral surface of either the atlas or axis for accurate drilling (C). A custom-made titanium plate was fixed to the realigned atlas and axis with cortical screws (D). Postoperative CT images (E, reconstructed sagittal plane; F, transverse plane). The luxated atlantoaxial joint was reduced and fixed to the titanium plate. The left lateral screw, shown on the right side in F, was inserted more laterally than planned.

After the diagnosis of AAI by CT and MRI (Fig 4A and 4B), drill guide templates and titanium plates were designed and fabricated as described. The drill guide template was held with a mosquito clamp and firmly pressed against the ventral surface of either the atlas or axis and drilling was performed through the sleeve (Fig 4C). A custom-made titanium plate was fixed to the realigned atlas and axis with a total of 7 cortical screws (Fig 4D). Postoperative CT images were acquired to confirm the anatomical relationship of the atlas and axis, as well as the locations of the inserted screws (Fig 4E and 4F).

### Accuracy of screw locations

Three screw holes (the center hole of C1 and cranial two holes of the axis) were drilled using the drill guide templates. The titanium plate was subsequently placed and fixed with cortical screws with a diameter of 1.2 mm or 1.7 mm depending on the size of the target vertebra. After placement of the titanium plate, the remaining screw holes were directly drilled through the plate screw openings and cortical screws were inserted. Therefore, the accuracy of the screw locations in each case was evaluated for 3 locations in which the screws were placed using the drill guide temples and 4 locations in which the screws were placed directly through the titanium plate without the use of the drill guide templates. Two cases were excluded from analyses because pre- and post-operative CT data were not obtained under the same imaging settings (Dog No.4 and 18). Deviations in the entry point for the C1 center screw on the X dimension (mediolateral deviation), Y dimension (dorsoventral deviation), and Z dimension (craniocaudal deviation) were 0.46 ± 0.31 mm, 0.41 ± 0.30 mm, and 0.69 ± 0.49 mm, respectively. Deviations in the exit point for the C1 center screw on the X, Y, and Z dimensions were 0.38 ± 0.29 mm, 0.60 ± 0.46 mm, and 0.99 ± 0.53 mm, respectively. Deviations in the entry point (both sides combined) for the cranial C2 screws on the X, Y, and Z dimensions were 0.62 ± 0.47 mm, 0.47 ± 0.38 mm, and 0.80 ± 0.51 mm, respectively. Deviations in the exit point for the cranial C2 screws on the X, Y, and Z dimensions were 0.93 ± 0.65 mm, 0.75 ± 0.59 mm, and 0.66 ± 0.44 mm, respectively. The lateral screws in C1 and caudal screws in C2 were placed without the use of the drill guide temples. The degree of deviations in these screws was similar to those placed with the use of the drill guide temples. Overall, the average ± SD deviations for all C1 entry points and exit points were 0.51 ± 0.51 mm and 0.82 ± 0.62 mm, respectively. The average ± SD deviations for all C2 entry points and exit points were 0.72 ± 0.61 mm and 0.72 ± 0.55 mm, respectively. Specific data for all screw deviations were summarized in Table 2.

**Table 2:** Specific data of deviations in screw entry points and exit points for the atlas and axis.

	C1 center						
		entry point				exit point	
	x	y	z		x	y	z
mean	0.46	0.41	0.69		0.38	0.6	0.99
sd	0.31	0.3	0.49		0.29	0.46	0.53
max	1.26	0.91	2.2		1.06	1.23	2.18
min	0.04	0.03	0.12		0.01	0.02	0.22
	C1 right						
		entry point				exit point	
	x	y	z		x	y	z
mean	0.4	0.47	0.91		0.94	1.04	1.09
sd	0.37	0.78	0.77		0.76	0.87	0.81
max	1.43	3.19	3.13		2.94	3.6	3.13
min	0.05	0	0.08		0.04	0.03	0.05
	C1 left						
		entry point				exit point	
	x	y	z		x	y	z
mean	0.38	0.42	0.5		0.64	0.78	0.9
sd	0.21	0.38	0.51		0.39	0.5	0.51
max	0.7	1.33	2.25		1.51	1.87	2.22
min	0.02	0.01	0.04		0.15	0.1	0.2
	C2 cranial right						
		entry point				exit point	
	x	y	z		x	y	z
mean	0.71	0.53	0.87		1.13	1.48	0.71
sd	0.53	0.44	0.55		0.75	2.73	0.46
max	1.66	1.85	1.84		2.92	2.6	1.75
min	0	0.14	0.03		0.05	0.05	0.14
	C2 cranial left						
		entry point				exit point	
	x	y	z		x	y	z
mean	0.53	0.41	0.74		0.72	1.91	0.62
sd	0.39	0.31	0.49		0.46	3.45	0.44
max	1.17	1.3	1.6		1.95	1.43	1.47
min	0.03	0.14	0.03		0.17	0.02	0.11
	C2 caudal right						
		entry point				exit point	
	x	y	z		x	y	z
mean	0.66	0.85	0.77		0.64	0.59	0.55
sd	0.52	0.81	0.65		0.49	0.41	0.63
max	1.63	3.16	2.54		1.71	1.4	2.5
min	0.03	0.11	0.07		0.04	0.07	0.03
	C2 caudal left						
		entry point				exit point	
	x	y	z		x	y	z
mean	0.98	0.57	0.87		0.92	0.61	0.71
sd	1.09	0.48	0.67		0.68	0.35	0.53
max	4.42	1.43	3.01		2.7	1.57	2.18
min	0.05	0.01	0.08		0.09	0.05	0.03

The X, Y, and Z dimensions represent mediolateral, dorsoventral, and craniocaudal deviations, respectively. All data are presented as the distance (mm) between the planned location and actual location.

### Intraoperative complications

The incidence of intraoperative complications resulting from screw insertion was assessed by intraoperative observations and postoperative CT images. No major complications occurred during the surgery. Intraoperative hemorrhage from the internal vertebral venous plexus was encountered in three cases when screw holes were drilled into the caudal axis and unintentionally penetrated the transcortex. Hemostasis was easily achieved by inserting a small volume of a hemostatic agent (SURGICEL®, Johnson & Johnson, Tokyo, Japan) and placing screws in the drill holes. Other than the above three cases, the violation of vital anatomical structures did not occur.

### Perioperative follow-up period outcomes

**Fig 5:**
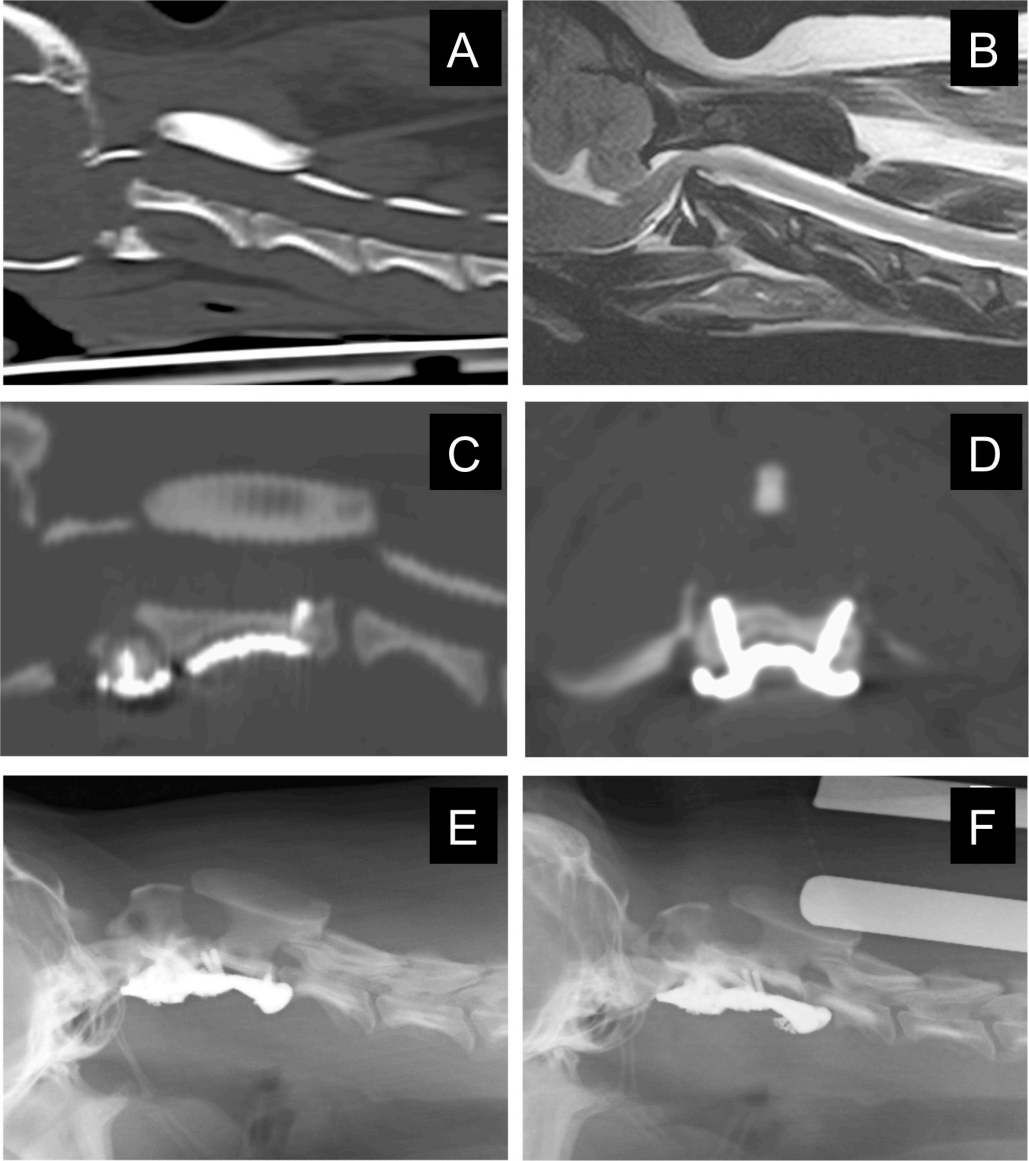
A case with C2 screw loosening (Case No.18). Preoperative CT (A) and MR (B) images demonstrating AA subluxation and spinal cord compression by the dorsally deviated axis. The dens appeared to be non-union. An immediate postoperative reconstructed sagittal image (C) and axial image (D). Screws in the atlas and axis are placed as planned and the luxated atlantoaxial joint was reduced. A postoperative radiograph obtained 33 days after surgery (E). The screws placed in C2 were loosened. A postoperative radiograph obtained 60 days after surgery (F). Further displacement of the C2 screws and dislocation of the AA joint were observed. This case underwent second surgery 91 days after the first surgery.

In the first visit, neurological deficits in most dogs were considered to be moderate to severe; the median neurological grade was 3 (range, 1–5). The conditions of 13 dogs (72.2%) improved after the application of a neck brace, and a low dose of prednisolone in 7 cases with the neurological grade of 1 or 2 at the first visit, with the median improvement of 2 grade (range, 1–3) in a re-examination on the day of surgery (Table 1). The conditions of the remaining 5 dogs were unchanged; these dogs presented with mild clinical signs in the first visit (1 dog with grade 3, 3 dogs with grade 4, 1 dog with grade 5). In the perioperative follow-up period assessed 2 weeks after surgery (n = 18), 7 dogs achieved further improvements, 10 dogs remained at the same grade, and 1 dog deteriorated from the pre-surgery status. Plain radiographs taken at these intervals demonstrated screw loosening in 6 dogs. In 5 dogs, screw loosening was detected in one of the C1 screws. In the remaining dog, the caudal C2 screws were almost completely pulled out and the cranial C2 screws were half way out (Fig 5). In the 6 dogs with screw loosening, the neurological grades between the pre-surgery and perioperative follow-up periods were unchanged in 4 dogs and improved in 2 dogs (Table 1). In order to assess the predisposing factors of screw loosening found in the 6 dogs, age, body weight, and neurological grades on the day of surgery were statistically compared against those of dogs without screw loosening. The age (mean ± SD) of dogs with screw loosening (56.8 ± 37.8 month) was not significantly different from that of dogs without screw loosening (34.7 ± 25.4 month) (*p* = 0.3024). Body weights (2.53 ± 0.59 kg and 2.71 ± 1.37 kg, respectively) (*p* = 0.7786) and neurological grades (3.8 ± 0.4 and 4.3 ± 0.7, respectively) (*p* = 0.49) were also not significantly different between the two groups. Dislocated screws may have been inserted into suboptimal locations; therefore, we statistically investigated the degree of deviations in the inserted screws from their planned coordinates. Deviations (distance shown in mean ± SD mm) in the entry points for C1 in dogs with screw loosening (0.86 ± 0.47) were not significantly different from those in dogs without screw loosening (1.07 ± 0.49) (*p* = 0.5847), and this was also the case for deviations in exit points for C1 in dogs with screw loosening (0.93 ± 0.24) and those without screw loosening (1.48 ± 0.49) (*p* = 0.0452).

Vertebral BMD and cortical thickness are predictors of screw pull-out strength [13, 14]. Therefore, we statistically compared the vertebral HU, as a representation of BMD, of C1 between dogs with and without screw loosening. The average HU of C1 in dogs with and without screw loosening were 1072 ± 184 HU and 983 ± 172 HU, respectively. The average cortical thickness of C1 in dogs with and without screw loosening were 3.38 ± 0.27 mm and 3.43 ± 0.62 mm, respectively. Comparisons of C1 vertebral HU (*p =* 0.3648) and cortical thickness (*p* = 0.8638) between dogs with and without screw loosening revealed no significant differences.

### Short follow-up outcomes

Short follow-up outcomes were assessed in a re-examination 3 months after surgery (n = 17). The median neurological grade was 5 (range, 4–5). In 5 dogs, the neurological grade improved from the 2-week post-surgery evaluation and 12 dogs remained at the same grade. None of the dogs deteriorated from pre-surgery or from the perioperative follow-up evaluations. Additional implant failure from the 2-week post-surgery evaluation was not detected in any of the dogs. One dog (Case No.17) was lost to the follow-up 9 months after surgery.

### Mid follow-up outcomes

Mid follow-up outcomes were assessed in a re-examination 12 months after surgery (n = 16). The median neurological grade was 5 (range, 4–5). One dog (Case No.7) died 11 months after surgery from an unknown reason. Although the C1 screw loosening was noticed in this dog at the perioperative period, the neurological condition of the dog improved during the short follow-up period and remained good until a sudden deterioration in its general condition when the dog presented with repetitive hematemesis which was not considered to be associated with neurologic diseases. The cause of death was not suspected to be related to the surgery performed; however, the definite cause was not identified because necropsy was not performed. Besides this case, the neurological grades of other dogs remained the same as those in the 3-month post-surgery evaluation. Case No.18, of which C2 screws were dislocated at the perioperative follow-up period, underwent second surgery 91 days after the first surgery due to dislocation of the plate even though the dog was clinically normal throughout (Fig 5). Revision surgery was performed with cortical screws, Kirschner wires, and PMMA after removing the titanium plate and screws placed in the first surgery. Despite screw loosening at C1 in the perioperative follow-up period in 5 dogs, none had plate dislocation and the AA joint appeared to remain intact.

## Discussion

We successfully utilized 3D printing technology, which enabled the manufacture of patient-specific drill guide templates and osteosynthesis plates to stabilize the AA joint in small breed dogs. The custom-made drill guide template provided accurate and safe guidance for screw placement by which the customized osteosynthesis plate was secured to the reduced AA joint. The smallest size among the cases treated was a 1.3-kg Toy Poodle. Even with the markedly small sizes of the target structures in this dog, reduction and stabilization were safely performed. Among the 18 dogs that underwent surgery, 15 (83.3%) exhibited clinical benefits at the mid-term follow up evaluation; therefore, the success rate of our surgical technique was at least similar, if not superior, to those of previous studies utilizing other surgical techniques for AAI stabilization [3, 4, 7, 10]. Similar technology has already been investigated in the area of orthopedics [15] [16], oral-facial surgery [17–19], and neurosurgery [20, 21] for the patient-specific manufacture of surgery guides and implants. In veterinary literature, investigation of patient-specific 3D printed drill guide for placement of cervical transpedicular screws was reported most recently [22].

In the present study, average deviations in the screws inserted into C1 and C2 were less than 1 mm. Drill guide templates allowed for the accurate guidance of screws and successfully aided in avoiding iatrogenic injuries to vital anatomical structures, which is of clinical importance. The only intraoperative complication encountered was hemorrhage from the internal vertebral venous plexus in three cases. This complication occurred because we unintentionally penetrated the distal cortex of the C2 and was not related to the misdirection of the drill hole. Therefore, the accuracy of our drill guide template system was considered to be clinically acceptable for the safe guidance of screw placement for AA fixation in dogs. In the present study, we estimated the accuracy of drill guide system by measuring the coordinate deviations of the entry point and exit point between pre- and post-operative CT images. In order to more precisely evaluate the accuracy of our system, the deviation of the screw trajectory needs to be evaluated in a three-dimensional manner in future study.

In human medicine, patient-specific drill guide templates have been developed as an inexpensive, accurate method to guide spinal fixation screws. The concept of personalized image-based 3D templates for spinal surgery was first described by Berry et al. [23], Owen et al. and Ryken and colleagues followed the concept with their cadaveric studies [24–26], and Lu and colleagues [27–29] and Kawaguchi et al. [30] then applied templates for clinical use as drill guide templates. Previous studies mostly used the DICOM data of CT images and computer software to generate plastic drill guide templates; however, misplacement occurred in up to 16% of pedicle screw insertions [24, 27, 31, 32]. Most drill guide templates were designed to fit the patient laminae, but were not secured on the laminae, which may have been one reason for the inaccuracy of the procedure [21]. Therefore, we designed templates to cover the 3D shape of the ventral surfaces of C1 and C2, and, accordingly, the templates were firmly attached to the bone. In addition, this 3D shape of the templates ensures that the procedure cannot be affected by spinal alignment changes, such as torsion during drilling and screw placement. Temporal fixation of the drill guide template to the bone by pins may prevent dislocation of the template; however, additional placement of pins may not be possible in small breed dogs.

Serious intraoperative and perioperative complications were not encountered in our cases. Perioperative and postoperative mortality rate is unknown with AA fixation surgery, but it has been reported that caudal brainstem trauma may be related to respiratory abnormalities and cardiac arrest in atlantoaxial subluxation repair [4]. Postoperative mortality may occur within 48 hours of surgery due to potential brainstem trauma causing damage to laryngeal and pharyngeal functions, which lead to the development of aspiration pneumonia [10]. Compression of the upper respiratory tract and laryngeal region by PMMA also impairs respiratory and swallowing function, which is a disadvantage of using PMMA in small breed dogs. The major postoperative complication in the present study was screw loosening in 6 dogs. Based on our analyses, age, body weight, and neurological grades at the time of surgery were not predisposing factors for screw loosening. The misplacement of screws was another possibility responsible for screw loosening; however, based on the measurement of screw corridor deviations the degree of screw mislocation was not significantly different between dogs with and without screw loosening. Therefore, the misplacement of screws was not considered to be related to screw loosening in our cases. The quality of bone such as BMD and cortical thickness are well-known factors associated with screw pull-out strength [13, 14]. In the present study, neither vertebral BMD nor cortical thickness appeared to predispose 6 dogs to screw loosening. Therefore, we considered the primary sources of screw loosening are accounted for by factors other than the inherent characteristics of patients themselves (case signalment, bone characteristics, etc) or changes in the screw-bone interface due to screw misplacement; they may be more closely associated with the design of the implants. The use of a locking screw and plate system was reported as an alternative to conventional PMMA-based techniques for AA stabilization [8]. The same technique may be incorporated into our patient-specific plate to improve the screw-plate interface, which is expected to markedly decrease the incidence of early screw back out.

We consider patient-specific osteosynthesis to provide several benefits clinically. For example, prefabricated plates are already an ideal shape, and, thus, intraoperative shaping and bending are avoided, which significantly reduces the surgical time [33]. Surgeons may use custom-made osteosynthesis plates as a “mold” to which the dislocated atlas and axis are simply attached, resulting in the planned anatomical relationship. In our cases, reductions in the atlas and axis and fixation to the plate did not require a significant effort. Although the technique presented in the present study provides significant improvements over currently available surgical methods for AAI, the lack of a control group limits precise comparison between our technique and previously described surgical techniques. There are limitations that need to be considered. This method requires time for design and fabrication. This limits the application of this technology to emergent cases. In the present study, some cases had a significantly long period of time between diagnosis and surgery. This long time period was due to all procedures being performed as research, and, thus, will markedly decrease when it becomes commercial. The design of screw corridors as well as the drill guide template and titanium plate and their fabrication may be completed within 4 to 5 days. Another limitation is that changes in the shapes of the bones to be fixed will result in suboptimal contact to the plate. This condition may be encountered with the development of degenerative bony changes or new bone formation in the target bones. Changes in the bone configuration may also occur in immature animals. In the present study, we encountered two cases that were 5 months old at presentation. CT imaging was delayed until 8 months old, at which point CT data was obtained for the design of the drill guide template and titanium plate. In these cases, contact between the plate and bone was still incomplete at the time of surgery because of changes in the bone configuration due to bone growth. Even under these conditions, fixation of the AA joint was feasible without significant technical difficulties. In both dogs, an improved neurological condition was confirmed in the long-term follow-up; therefore, clinical benefits were achieved, even under these suboptimal conditions.

## Conclusion

We herein demonstrated the successful application of 3D printing technology to the surgical treatment of AAI in small breed dogs. Our drill guide templates allowed for the accurate placement of cortical screws in substantially small target bones and patient-specific titanium plates effectively stabilized the AA joint without any intraoperative major complications and apparent implant-related mortality. The efficacy of the combination of a similar intraoperative guide device and custom-made osteosynthesis plate indicates its potential for application to the surgical treatment of other spinal disorders in dogs.
